# Cholesterol esterification inhibition and gemcitabine synergistically suppress pancreatic ductal adenocarcinoma proliferation

**DOI:** 10.1371/journal.pone.0193318

**Published:** 2018-02-28

**Authors:** Junjie Li, Xiaochao Qu, Jie Tian, Jian-Ting Zhang, Ji-Xin Cheng

**Affiliations:** 1 Weldon School of Biomedical Engineering, Purdue University, West Lafayette, Indiana, United States of America; 2 School of Life Science and Technology, Xidian University, Xi’an, Shaanxi, China; 3 Institute of Automation, Chinese Academy of Sciences, Beijing, China; 4 Department of Pharmacology and Toxicology, Indiana University School of Medicine, Indianapolis, Indiana, United States of America; 5 Center for Cancer Research, Purdue University, West Lafayette, Indiana, United States of America; 6 Department of Biomedical Engineering, Department of Electrical and Computer Engineering, Boston University, Boston, Massachusetts, United States of America; University of South Alabama Mitchell Cancer Institute, UNITED STATES

## Abstract

Recent advances have recognized metabolic reprogramming as an underlying mechanism for cancer drug resistance. However, the role of cholesterol metabolism in drug resistance remain elusive. Herein, we report an increased accumulation of cholesteryl ester in gemcitabine-resistant pancreatic ductal adenocarcinoma (PDAC) cells. A potent inhibitor of acyl-CoA cholesterol acyltransferase-1 (ACAT-1), avasimibe, effectively suppressed proliferation of gemcitabine-resistant PDAC cells. Combination of avasimibe and gemcitabine showed strong synergistic effect in suppressing PDAC cell viability *in vitro* and tumor growth *in vivo*. Immunoblotting analysis suggests downregulation of Akt by avasimibe is likely to contribute to the synergism. Collectively, our study demonstrates a new combinational therapeutic strategy to overcome gemcitabine resistance for PDAC treatment.

## Introduction

Drug resistance is one of the most challenging problems that hamper the success of complete cancer treatment [1,2]. Compared to the primary resistance, which exists prior to any treatment, acquired resistance represents more challenges for cancer therapy [1]. Extensive studies have deciphered some key mechanisms underlying acquired drug resistance, such as drug inactivation, target alteration, induction of alternative pro-survival pathways, and existence of cancer stem cells [3,4]. Recent evidences further link metabolic alterations to drug resistance in cancer cells. Targeting the reprogrammed metabolism is emerging as a novel strategy to beat the cancer drug resistance [5,6]. Studies have found that targeting glycolytic pathway overcomes resistance to chemotherapies, such as trastuzumab and taxol, in breast cancer [7,8]. Similarly, overexpression of pyruvate dehydrogenase kinase 3 (PDK3) promotes a metabolic switch from mitochondrial respiration to glycolysis under hypoxia condition and increases drug resistance in cervical and colon cancer [9,10]. Besides, fatty acid synthase (FASN), a key enzyme in the fatty acid synthesis pathway, was reported to be involved in multiple drug resistance, such as docetaxel/trastuzumab resistance in breast cancer and gemcitabine or radiation resistance in pancreatic cancer [11–15]. Despite these advances, cholesterol metabolism remains an underexplored area in terms of drug resistance in cancers.

Recent studies have identified an aberrant accumulation of cholesteryl ester (CE) mediated by the enzyme Acyl coenzyme A: cholesterol acyltransferase 1 (ACAT-1) in multiple cancers, including prostate, pancreatic, leukemia, glioma, breast, colon and lung cancer [16–22]. Inhibition of ACAT-1 using a small molecule inhibitor, avasimibe, effectively removed CE accumulation and suppressed tumor growth. Accumulation of CE was further found to be driven by activation of the PI3K/Akt signaling pathway, which often occurs in advanced-stage cancers, as well as in drug-resistant cancer cells. Here, using Raman spectroscopic imaging techniques, we identified a higher level of CE accumulation in a treatment-induced gemcitabine-resistant pancreatic ductal adenocarcinoma (PDAC) cell line, when compared to gemcitabine sensitive parental cells. Avasimibe effectively suppresses cell viability in gemcitabine-resistant cells and shows strong synergistic effects with gemcitabine. The synergy was further validated in a xenograft mouse model of PDAC. Mechanistically, we show that avasimibe overcomes gemcitabine resistance by downregulating the Akt signaling pathway. These results collectively suggest a novel therapeutic strategy for treating gemcitabine-resistant PDAC.

## Results

### CE accumulation in gemcitabine-resistant PDAC cells

**Fig 1:**
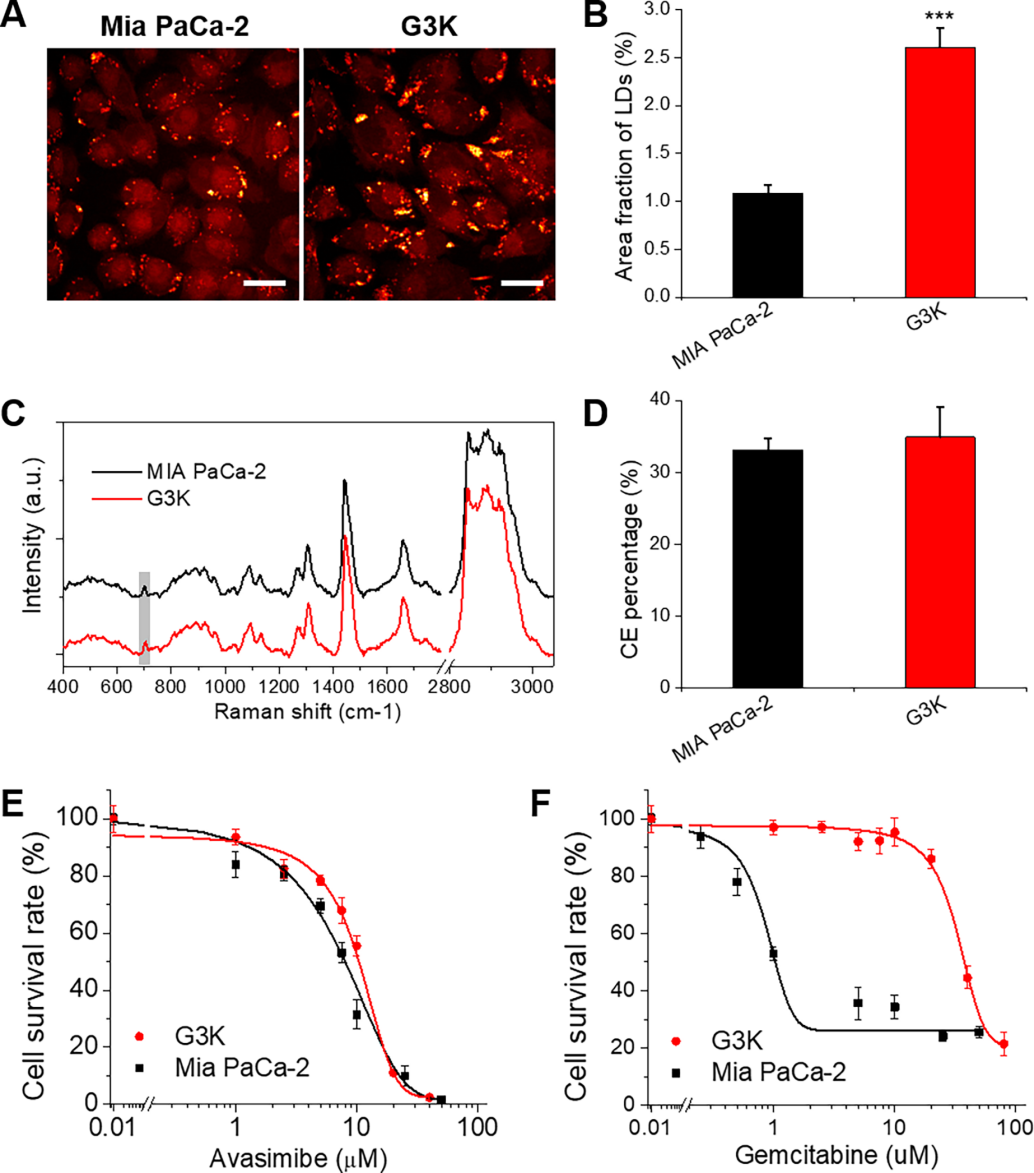
An increased CE accumulation was found in gemcitabine resistant pancreatic cancer cells compared to parental cells. (**A**) Representative SRS images of gemcitabine-sensitive Mia PaCa-2 cells and gemcitabine-resistant G3K cells. Scale bar: 10 μm. (**B**) Quantification of LD amount in Mia PaCa-2 and G3K cells. The results are shown as means + SD, n = 6, *** *p* < 0.001. (**C**) Representative Raman spectra taken from individual LDs in Mia PaCa-2 and G3K cells. The spectra were offset to clarify the peak. The peak at 702 cm^-1^ as a characteristic peak of cholesterol was highlighted in gray shade. (**D**) Quantification of CE percentage out of total lipids in LDs in Mia PaCa-2 and G3K cells. The results are shown as means + SD, n ≥ 10. (**E, F**) Cell viability assay of Mia PaCa-2 and G3K cells treated with (E) avasimibe or (F) gemcitabine at indicated concentrations. The experimental data points were fitted using Dose-Response function in Origin 8.5 software. The results are shown as means ± SD, n = 6.

To study the lipid metabolism in acquired gemcitabine-resistant PDAC cells, we generated a gemcitabine-resistant G3K cell line by continuous gemcitabine selection in Mia PaCa-2 cells as described earlier [13]. Stimulated Raman Scattering (SRS) imaging was used to assess the lipid amount in Mia PaCa-2 and G3K cells by tuning the laser beating frequency to match the vibrational frequency of C-H bond. SRS images show more aggregation of lipid droplets (LDs) in G3K cells, compared to Mia PaCa-2 cells (**Fig 1A**). Quantification of LDs area out of total cellular area, as a measurement of lipid amount, reveales a significant increase of lipid amount in G3K cells than Mia PaCa-2 cells (**Fig 1B**). To further analyze the composition of the lipids inside LDs, we acquired Raman spectra from LDs in Mia PaCa-2 and G3K cells (**Fig 1C**), which confirm the presence of cholesteryl ester (CE) in LDs of both cell lines, as evidenced by the peak at ~700 cm^-1^. Through a quantitative analysis following the protocol established in our previous study [17], we found that LDs in both cell lines contain relatively high level of CE (**Fig 1D**). Considering the largely increased lipid amount in G3K cells, the total amount of CE in G3K cells is much higher than Mia PaCa-2 cells, suggesting a possibility to treat gemcitabine-resistant G3K cells by targeting cholesterol esterification pathway.

Then we applied an inhibitor of ACAT-1, avasimibe, and tested its efficacy in Mia PaCa-2 and G3K cells. The results show that avasimibe effectively suppresses cell viability of both Mia PaCa-2 and G3K cells with IC_50_s of 7.0 and 8.85 μM, respectively (**Fig 1E**). In contrast, the IC_50_s of gemcitabine in Mia PaCa-2 and G3K cells are 1.23 and 36.34 μM, respectively (**Fig 1F**), indicating G3K cells are highly resistant to gemcitabine. These results show a high antitumor effectiveness of avasimibe even in gemcitabine resistant cancer cells.

### Combination of avasimibe and gemcitabine shows synergistic effect *in vitro*

**Fig 2:**
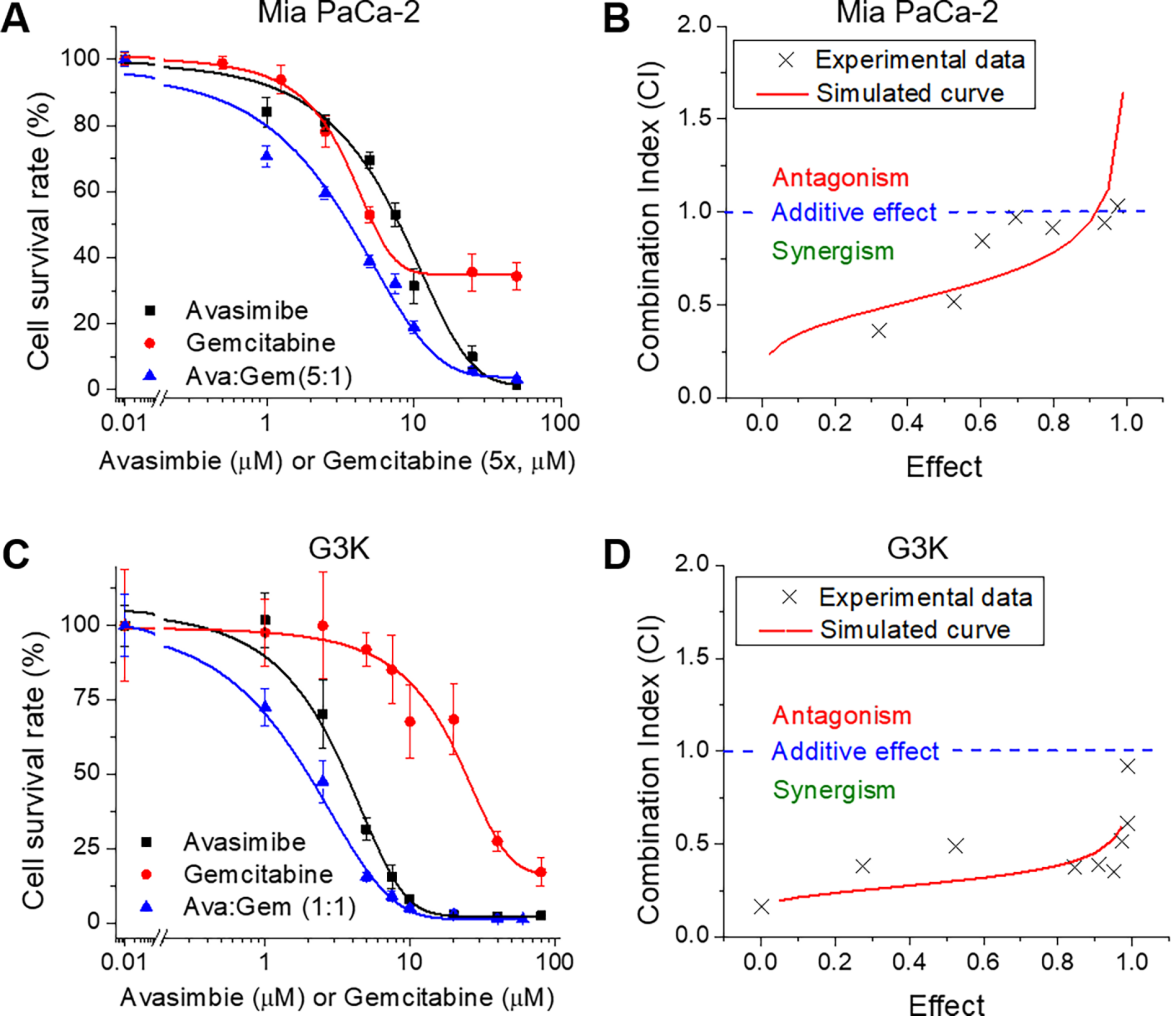
Avasimibe and gemcitabine show synergistic effect in suppressing cell viability *in vitro*. (**A**) Cell survival assay of Mia PaCa-2 cells treated with avasimibe, gemcitabine or combination of avasimibe and gemcitabine at a molar ratio of 5:1 (Ava:Gem) for 3 days. Gemcitabine was plotted as 5×dose. (**B**) Plot of CI versus fractional effect using the data shown in (A). (**C**) Cell survival assay of G3K cells treated with avasimibe, gemcitabine or combination of both at a molar ratio of 1:1 for 3 days. (**D**) Plot of CI versus fractional effect using data shown in (C). For cell viability results, the data points were fitted by Dose-Response function using Origin 8.5 software. The results are shown as means ± SD, n = 6. For CI plots, crosses indicate experimental data from the combinational treatment. Curves with solid lines are simulated CI/fractional effect plots. CI < 1.0 indicates synergism; CI = 1.0 indicates additive effect; and CI > 1.0 indicates antagonism.

Having shown the efficacy of avasimibe in gemcitabine-resistant cells, we further explored the possibilities of combining gemcitabine with avasimibe. Combinations of gemcitabine and avasimibe at various ratios were firstly tested in Mia PaCa-2 cells and compared with the effects of single drug treatments. To determine if a synergistic effect exists between avasimibe and gemcitabine, we analyzed the data following Chou–Talalay method [23]. Combination index (CI) and dose reduction index (DRI) were calculated using CalcuSyn software. At a molar concentration ratio of 5:1 (avasimibe: gemcitabine), the combinational therapy exhibits a better inhibitory effects on cell viability, compared to single agent treatments (**Fig 2A**). Plot of the calculated CI values versus the inhibitory fractional effects (0–1) shows most CIs are smaller than 1.0, indicating synergism between avasimibe and gemcitabine (**Fig 2B**). Synergism was also observed in combinations at various molar concentration ratios, including 10:1 and 15:1 (avasimibe: gemcitabine), at almost all inhibitory doses (**Table 1**). Next, we further tested the synergistic effect in gemcitabine-resistant G3K cells. The results show a better anti-proliferation effect in G3K cells when avasimibe and gemcitabine was combined at a molar ratio of 1:1, compared to the single-drug treatments (**Fig 2C**). The CI values are mostly below 0.5, suggesting a stronger synergism between avasimibe and gemcitabine in G3K cells than in Mia PaCa-2 cells (**Fig 2D**).

**Table 1:** Synergistic effects of avasimibe and gemcitabine in Mia PaCa2 cells combined at various molar concentration ratios.

Drug combination	CI value at				DRI value at				
	*IC*_30_	*IC*_50_	*IC*_75_	*IC*_90_		*IC*_30_	*IC*_50_	*IC*_75_	*IC*_90_
Ava + Gem (5:1)	0.487	0.597	0.779	1.017	Ava	2.491	2.045	1.583	1.226
					Gem	11.759	9.247	6.772	4.960
Ava + Gem (10:1)	0.469	0.550	0.676	0.831	Ava	2.357	2.020	1.653	1.353
					Gem	22.256	18.267	14.140	10.946
Ava + Gem (15:1)	0.497	0.567	0.672	0.797	Ava	2.152	1.893	1.603	1.357
					Gem	30.480	25.683	20.570	16.474

### Combinational therapy of avasimibe and gemcitabine induces tumor regression in xenograft mouse model of PDAC

**Fig 3:**
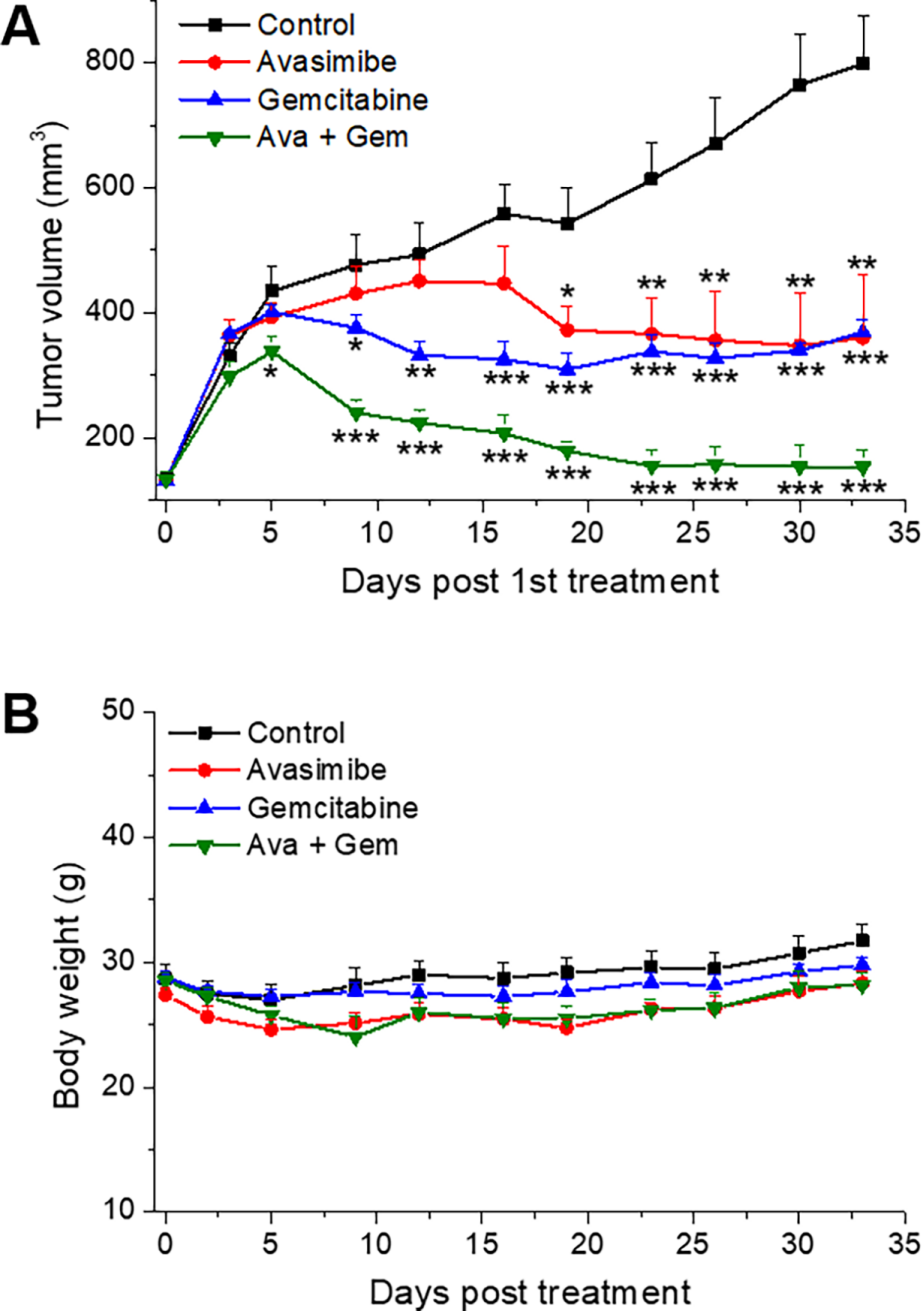
Combination of avasimibe and gemcitabine synergistically suppressed pancreatic tumor growth in vivo. (**A**) Tumor growth curves of mice treated with vehicle (control), avasimibe (Ava, 7.5 mg/kg), gemcitabine (Gem, 50 mg/kg), or combination of avasimibe and gemcitabine at the same dose (Ava + Gem). (**B**) Monitoring of mice body weight during treatment. The results are shown as means + SD, n = 8, * *p* < 0.05, ** *p* < 0.01, *** *p* < 0.001.

To further validate the antitumor effect of the combination therapy *in vivo*, a subcutaneous xenograft mouse model derived from Mia PaCa-2 cells was established. Single treatment with avasimibe or gemcitabine shows effectiveness in reducing tumor growth rate and induces slight tumor remission after a period of treatment. Tumors grew up again after 20-days gemcitabine treatment, indicating the appearance of gemcitabine-resistance. Avasimibe treatment alone maintains tumor size at a constant level over the treatment period. In contrast, combinational therapy reverses tumor growth by substantially reducing tumor size with no sign of tumor recovery even after 34-days treatment (**Fig 3A**), indicating a nearly complete tumor remission was achieved by the combination therapy. Notably, no treatment associated body weight loss was observed when compared to single treatment groups (**Fig 3B**). These data demonstrate a much-enhanced anti-cancer effects of avasimibe and gemcitabine when administrated together as a combinational therapy.

### Avasimibe overcomes gemcitabine-resistance by downregulating Akt pathway

**Fig 4:**
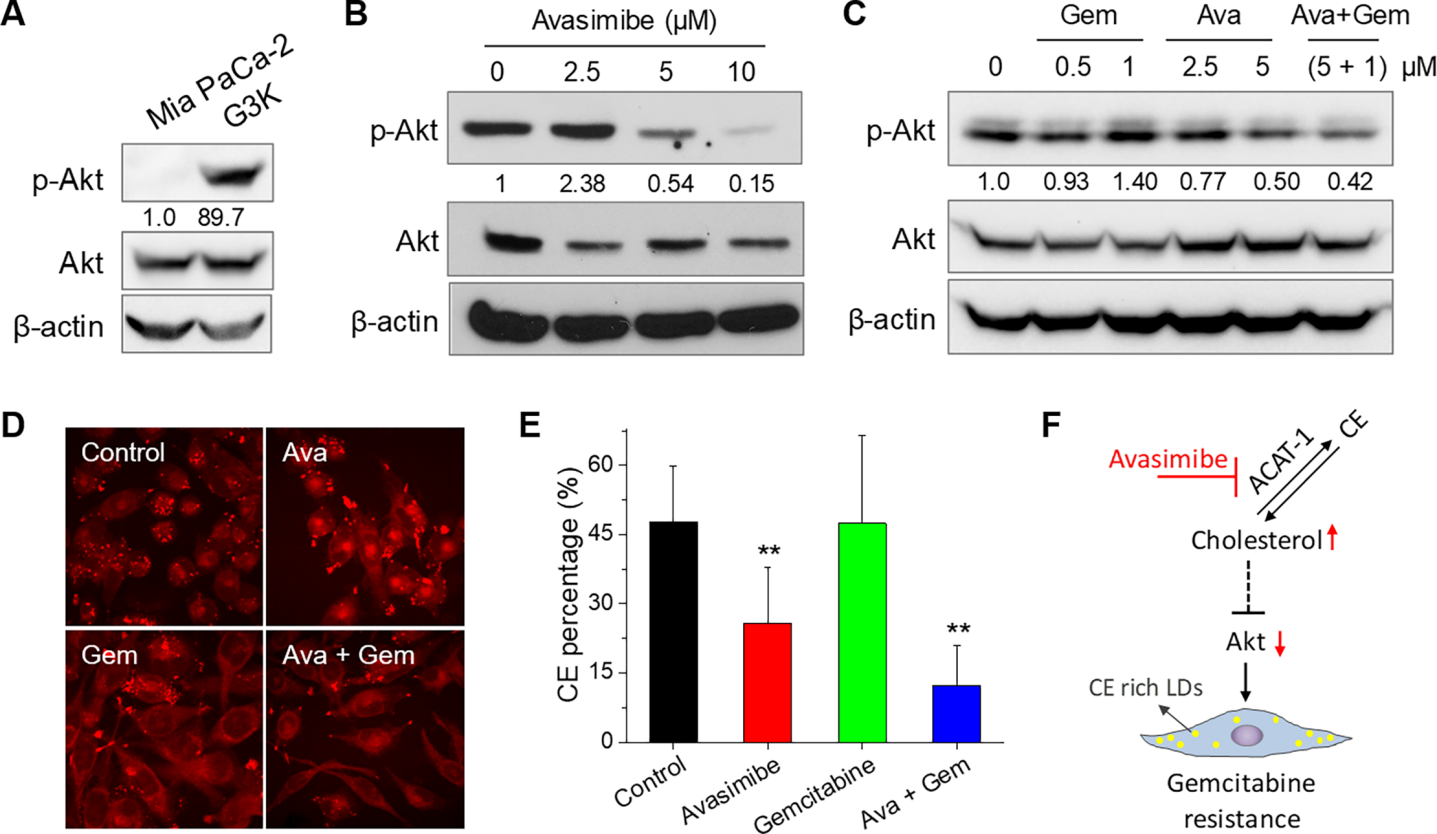
Avasimibe resensitizes pancreatic cancer cells to gemcitabine treatment by suppressing Akt activity. (**A**) Immunoblotting of β-actin, Akt, and p-Akt in Mia PaCa-2 and G3K cells. (**B**) Immunoblotting of β-actin, Akt, and p-Akt in G3K cells treated with avasimibe at indicated concentrations for 48 hours. (**C**) Representative SRS images of Mia PaCa-2 cells treated with avasimibe, gemcitabine, or combination of both for 48 hours. (**D**) Quantitative analysis of CE percentage of total lipid in Mia PaCa-2 cells with indicated treatments. The results are shown as means + SD, n ≥ 10, ** *p* < 0.01. (**E**) Immunoblotting of β-actin, Akt, and p-Akt in G3K cells under indicated conditions. For Western blot data, quantification of the ratios of p-Akt to Akt is shown below each p-Akt band. (**F**) Schematic drawing showing the mechanism how avasimibe overcomes gemcitabine resistance in pancreatic cancer.

To investigate the potential mechanisms by which avasimibe overcomes gemcitabine resistance, we have performed immunoblotting to evaluate the changes of key signaling pathways. As Akt pathway has been known as one of the key signaling pathways associated with gemcitabine-resistance in PDAC [24], we firstly examined the expression level of total Akt and phosphorylated-Akt in the gemcitabine-sensitive Mia PaCa-2 cells and gemcitabine-resistant G3K cells. An largely increased expression level of p-Akt was found in G3K cells, suggesting a correlation between Akt activity and gemcitabine-resistance (**Fig 4A**). We further found avasimibe treatment decreased the expression levels of p-Akt in a dose-dependent manner (**Fig 4B**). Consistent with gemcitabine-resistance, gemcitabine treatment increased expression level of p-Akt. Avasimibe treatment alone, or combined with gemcitabine at a molar concentration ratio of 5:1 (avasimibe: gemcitabine) significantly reduced the level of p-Akt (**Fig 4C**). Combination of avasimibe with gemcitabine also significantly reduced CE accumulation in Mia PaCa-2 cells, as evidenced by SRS images and Raman spectral analysis (**Fig 4D and 4E**), suggesting the downregulation of Akt signaling by avasimibe is associated with reduced CE level. The mechanism of how ACAT1 inhibition downregulates Akt signaling is likely to be mediated by the increased free cholesterol level [17], but further studies will be needed. In summary, as illustrated in Fig 4F, our data suggests that avasimibe overcomes gemcitabine-resistance by downregulating gemcitabine-resistance associated Akt signaling pathway, which is likely mediated by increased free cholesterol level.

## Discussion

Pancreatic cancer (mostly PDAC) remains the fourth leading cause of cancer death in 2018, with 55,440estimated new cases and 44,330estimated new deaths [25]. Since 1996, gemcitabine has been used as the cornerstone for treating this deadly disease, despite its modest overall effects to the patients. Development of resistance to gemcitabine in almost 100% of the patients further hampers its clinical benefits. To develop an effective therapeutic strategy to target gemcitabine resistance represents an unmet need in PDAC treatment. In this study, for the first time we showed that cholesterol metabolism is related to gemcitabine resistance in PDAC. Inhibitor of cholesterol esterification, avasimibe, synergistically suppresses PDAC cell proliferation with gemcitabine, suggesting it as a potential anti-cancer agent for aggressive PDAC treatment.

Using SRS imaging and Raman spectroscopy, we found a higher level of CE accumulation in gemcitabine-resistant PDAC cells than the parental gemcitabine-sensitive cells. However, how CE accumulation contributes to gemcitabine-resistance remains elusive. In our previous studies, we have shown that CE accumulation in cancers is driven by the PI3K/Akt signaling pathway [16,17], which is also known to associate with gemcitabine-resistance in pancreatic cancer [26]. Our data supports an increased Akt activity in gemcitabine-resistant cells compared to gemcitabine-sensitive cells. It is likely that cancer cells convert free cholesterol into CE to maintain a relatively low level of free cholesterol in the ER, and thus to constitutively activate SREBP transcription factors. Inhibition of cholesterol esterification disturbs the cholesterol homeostasis, inactivates the Akt signaling possibly through feedback mechanism, and re-sensitize PDAC cells to gemcitabine treatment.

Beside the PI3K/Akt pathway, it is possible that inhibition of cholesterol esterification overcomes drug resistance through other mechanisms. In a recent study, cholesterol metabolism was implicated in the development of resistance to tamoxifen in breast cancer [27]. CE, along with some other cholesterol metabolites, was shown to promote cancer aggressiveness [28,29]. Therefore, cholesterol esterification is likely to contribute to the development of gemcitabine-resistance through undetermined mechanisms. For example, caveolin-dependent cholesterol efflux pathways have been linked to multiple drug resistance [30]. CE accumulation might be related to the upregulation of caveolin-1, which promotes the gemcitabine-resistance. Furthermore, it is worth noting that a few recent studies have reported an enhanced antitumor activity of immune system when cholesterol esterification is blocked [31,32]. ACAT1 inhibitors was shown to increase plasma membrane cholesterol level and promote proliferation of CD8^+^ T cells [32]. Combination of avasimibe with immunotherapy, such as anti-PD-1 antibody therapy or chimeric antigen receptor-modified T cell therapy, show enhanced antitumor effects [31,32]. These results suggest that immune response plays an important role in avasimibe’s antitumor effects, and possibly in overcoming gemcitabine resistance in pancreatic cancers. Future *in vivo* studies using spontaneous mouse models of pancreatic cancer will be needed to validate the efficacy of the combinational therapy of avasimibe and gemcitabine and to fully elucidate the mechanisms that link cholesterol metabolism and cancer drug resistance.

## Materials and methods

### Cell lines and chemicals

Human PDAC cell line Mia PaCa-2 was obtained from the American Type Culture Collection (ATCC). Gemcitabine-resistant G3K cell line was generated from parental Mia PaCa-2 cells by continuous culture with the presence of gemcitabine [13]. Mia PaCa-2 cells were cultured in RPMI 1640 medium supplemented with 10% FBS and 1% Penicillin/Streptomycin. G3K cells were cultured in the same media supplemented with 1.0 μM gemcitabine to maintain the resistance. For maintenance, all cells were cultured at 37°C in a humidified incubator with 5% CO_2_ supply. Chemicals including avasimibe, gemcitabine used *in vitro* and *in vivo* studies were purchased from Selleckchem.com.

### Cell viability assay

Cell viability was measured by Thiazolyl Blue Tetrazolium Blue (MTT) colorimetric assay (Sigma). Cells were seeded at day 0 in 96-well plates at same density and incubated overnight. Treatments at indicated concentrations were added at day 1 and incubated for another three days. At day 4, MTT agents were added and absorbance was read at 570 nm with a plate-reader (Molecular Devices, SpectraMax M5).

### Drug combination analysis

Combination of avasimibe and gemcitabine was performed at various molar concentration ratios, including 5:1, 10:1 and 15:1 (ava: gem). To assess the synergism, the Combination Index (CI) was calculated by the Chou–Talalay equation [23], which takes into account both the potency (IC_50_) and shape of the dose–effect curve, determined by the CalcuSyn software (Biosoft, Cambridge, UK). The general equation for the classic isobologram is given by CI = (D)_1_/(Dx)_1_ + (D)_2_/(Dx)_2_ + [(D)_1_ · (D)_2_]/[(Dx)_1_ · (Dx)_2_], where (Dx)_1_ and (Dx)_2_ in the denominators are the doses (or concentrations) of D1 (drug 1) and D2 (drug 2) alone that induces *x*% growth inhibition, whereas (D)_1_ and (D)_2_ in the numerators are the doses (or concentrations) of drug 1 and drug 2 that in combination also inhibit *x*% cell growth (i.e., isoeffective). CI/fractional effect curves represent the CI versus the fraction (0 → 1) of cells killed by drug combinations. CI < 1 indicates synergism; CI = 1 indicates an additive effect; and CI > 1 indicates antagonism. The dose-reduction index (DRI) represents the extent (folds) of dose reduction in a combination, for a given degree of cell growth inhibition effect, compared to each drug alone. Throughout all experiments, we obtained a linear correlation coefficient of *r* > 0.90.

### In vivo study in xenograft mouse model

All animal experiments were conducted following protocols approved by Purdue Animal Care and Use Committee (PACUC). 4~6-week old female athymic nude mice (Envigo) were subcutaneously injected with ~5 × 10^6^ MIA PaCa-2 cells per mouse. Mice were anesthetized using isoflurane inhalation when tumor cells were injected. One week after tumor cell inoculation, the mice were divided into four groups with 8 mice in each group. These four groups will be treated with vehicle (DMSO), avasimibe (7.5 mg/kg in DMSO, daily), gemcitabine (50 mg/kg in PBS, once every 3 days), and combination of avasimibe (7.5 mg/kg in DMSO, daily) and gemcitabine (50 mg/kg in PBS, once every 3 days). All the treatments were administrated by intraperitoneal injection. Tumor volume and body weight were measured twice every week. Tumor volume was calculated following the formula volume = 1/2 × length × width^2^. Treatment was discontinued when the tumor volume reached 2000 mm^3^ or when the tumor was interfering with movement, whichever came first. Mice were sacrificed by cervical dislocation following deep anesthesia induced by isoflurane inhalation, as approved by PACUC protocol. Every effort was made to minimize suffering and to reduce the number of animals necessary to complete the study.

### Stimulated Raman scattering (SRS) imaging and Raman spectroscopy

SRS imaging and Raman spectra acquisition were performed to cultured cells in glass-bottom dishes as previously described [17]. Lipid amount was quantified from SRS images using ImageJ software. LDs were picked up by applying a threshold and the area fraction of LDs out of total cellular area was calculated. For each group, measurements from 6 different areas were used for statistical analysis. Quantification of CE percentage out of total lipids in LDs were conducted by measuring the intensity ratio of the peak at 702 cm^-1^ to the peak at 1450 cm^-1^, which is linearly correlated to the molar fraction of CE as determined previously [17]. For statistical analysis, at least 10 spectra were analyzed for each group.

### Immunoblotting

Cells at indicated conditions were harvested and lysed in RIPA lysis buffer (Sigma-Aldrich) supplemented with protease and phosphatase inhibitor cocktails. Protein concentration was measured using the Bio-Rad Protein Assay kit. Protein extraction was subjected to immunoblotting with the antibodies against Akt (Cell Signaling, #4691), p-Akt (Cell Signaling, #4060L) and β-actin (Sigma, A5441). Antibody was diluted following manufacture’s recommendation (1:1000 for Akt/p-Akt, 1:5000 for β-actin). β-actin was used as loading control. Horseradish peroxidase (HRP) conjugated secondary antibody (BIO-RAD) was used at a dilution of 1:2000.

### Statistical analysis

Results were represented as means +/± SD or as specified. One-way ANOVA or t-test was used for statistical analysis. Statistical significance was indicated as * *P* < 0.05, ** *P* < 0.01, and *** *P* < 0.001.
